# Characterization of Low-Symmetry Structures from Phase Equilibrium of Fe-Al System—Microstructures and Mechanical Properties

**DOI:** 10.3390/ma8030914

**Published:** 2015-03-04

**Authors:** Piotr Matysik, Stanisław Jóźwiak, Tomasz Czujko

**Affiliations:** Department of Advanced Materials and Technologies, Faculty of Advanced Technologies and Chemistry, Military University of Technology, Gen. S. Kaliskiego 2 St., Warsaw 00-908, Poland; E-Mails: sjozwiak@wat.edu.pl (S.J.); tczujko@wat.edu.pl (T.C.)

**Keywords:** Al-Fe alloy, microstructure, mechanical properties, SEM/EDS, XRD, nano-indentation

## Abstract

Fe-Al intermetallic alloys with aluminum content over 60 at% are in the area of the phase equilibrium diagram that is considerably less investigated in comparison to the high-symmetry Fe_3_Al and FeAl phases. Ambiguous crystallographic information and incoherent data referring to the phase equilibrium diagrams placed in a high-aluminum range have caused confusions and misinformation. Nowadays unequivocal material properties description of FeAl_2_, Fe_2_Al_5_ and FeAl_3_ intermetallic alloys is still incomplete. In this paper, the influence of aluminum content and processing parameters on phase composition is presented. The occurrence of low-symmetry FeAl_2_, Fe_2_Al_5_ and FeAl_3_ structures determined by chemical composition and phase transformations was defined by scanning electron microscopy (SEM) and energy-dispersive X-ray spectroscopy (EDS) examinations. These results served to verify diffraction investigations (XRD) and to explain the mechanical properties of cast materials such as: hardness, Young’s modulus and fracture toughness evaluated using the nano-indentation technique.

## 1. Introduction

Iron aluminides are considered as structural and functional materials, increasingly used as intermetallic sinters [1,2,3,4,5,6,7,8,9,10], graded materials [11], or in the form of HVOF and gas detonation sprayed layers [12,13,14]. Unfortunately, it should be noted that the functional properties of these alloys, especially the mechanical properties, are determined only for the relatively plastic FeAl and Fe_3_Al phases and solid solution with aluminum content restricted to 50 at%. In the case of Al-rich phases from the Fe-Al system (aP18 FeAl_2_, oC14 Fe_2_Al_5_, and mC102 FeAl_3_), the area of their occurrence has not been well-defined yet. Despite to the research undertaken in order to complement or present a detailed description of their mechanical properties depending on manufacturing parameters, e.g., temperature, pressure and chemical composition [15,16,17,18,19,20,21,22], the presented results are often ambiguous and contradictory [7,10,23,24,25,26,27,28,29,30,31]. Ambiguities within determining the fields of structural stability of the Al-rich phases in the description of the Fe-Al system drastically reduce the potential use of these structures in the techniques of aluminum coating, soldering and welding [32,33,34] as well as sintering elementary iron and aluminum powders [35,36,37,38].

Therefore, in this paper we attempt to clarify the aluminum content ranges responsible for changes in the crystal structure of the analyzed Al-rich phases. The measurements of selected mechanical properties were made for polycrystalline disordered intermetallic alloys based on phases—Triclinic FeAl_2_, orthorhombic Fe_2_Al_5_ and monoclinic FeAl_3_ in which low crystal symmetry prevents the disorder—Order transformation. The authors pointed out the need for further research in this area, particularly that leading to clarify the phase transformation processes.

## 2. Results and Discussion

### 2.1. Structural Studies

**Figure 1:**
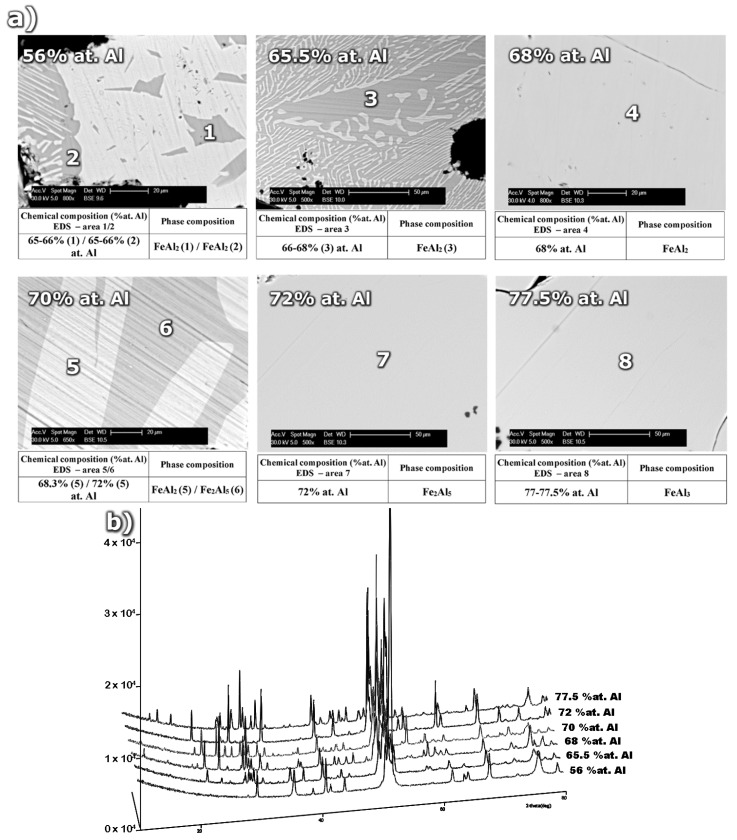
(**a**) The microstructure and (**b**) XRD phase analysis of homogenized sinters as a function of aluminum content.

The microstructure of samples with various aluminum content after sintering and annealing at 1200 °C/24 h is presented in Figure 1. The important differences in phase composition result from the stoichiometry of the structural components and phase transformations occurring during the sintering and homogenization processes. The samples with aluminum content in the range of 56.0–65.5 at% are characterized by a dual phase FeAl-FeAl_2_ structure determined by EDS examinations in micro areas. Moreover, the mixture of FeAl + FeAl_2_ crystals is observed. It results from the eutectoid reaction of the ε (Fe_5_Al_8_) phase decomposition at 1092 °C. A single phase homogeneous sinter which is composed of FeAl_2_ perytectoid grains (Figure 1b) was observed for the sample with aluminum content amounting to 68 at% (Figure 1).

**Figure 2:**
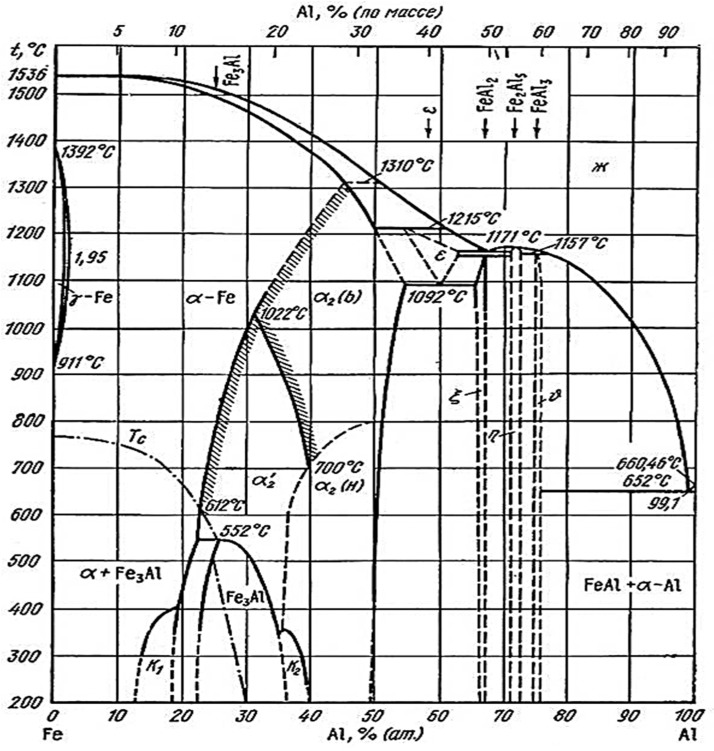
Fe-Al binary diagram proposed by Kubaschewski and approved during Discussion Meeting on the Development of Innovative Iron Aluminum Alloys, for analysis and phase transformation interpretation in iron-aluminum alloys. Ambiguous area of Al-rich phases occurrence were marked by dashed line [19].

The continuous precipitates within the intermetallic matrix visible in Figure 1a for the above chemical composition (68 at% Al) are identified as aluminum oxides. They are an integral structural element occurring during sintering process of mixtures containing strongly passivating aluminum powder. The increase of aluminum concentration from 68 at% Al which is a typical content for the ε + Fe_2_Al_5_↔FeAl_2_ peritectoid reaction to 70 at% results in the creation of a dual-phase structure. This structure consists of the FeAl_2_ phase, formed as a result of the peritectoid reaction, and the Fe_2_Al_5_ phase crystallized from liquid. The next, in terms of the increasing aluminum content, phase-homogeneous sinter made of the Fe_2_Al_5_ phase identified during XRD analysis (Figure 1b), was obtained for 72 at% Al (Figure 1a). The next, single-phase alloy of the Fe-Al system, produced using powder metallurgy, was obtained for 77.5 at% Al. This value of aluminum content in the structure of the single-phase homogenous alloy, identified as the FeAl_3_ phase, is slightly higher than the stoichiometry given by Kubaschewski (Figure 2) which equals to 75 at% Al. The noticed discrepancy requires additional confirmation but shows ambiguities not only in the interpretation of the content of the components constituting the phase data.

Nonetheless, the results obtained during the study on the content of aluminum in sintered materials, which allows the formation of homogeneous Al-rich intermetallic FeAl_2_, Fe_2_Al_5_ and FeAl_3_ phases, enabled the forming of alloys with anticipated crystalline structure by melting and vacuum casting. What is more important, obtained by these methods alloys are devoid of oxide precipitates.

**Figure 3:**
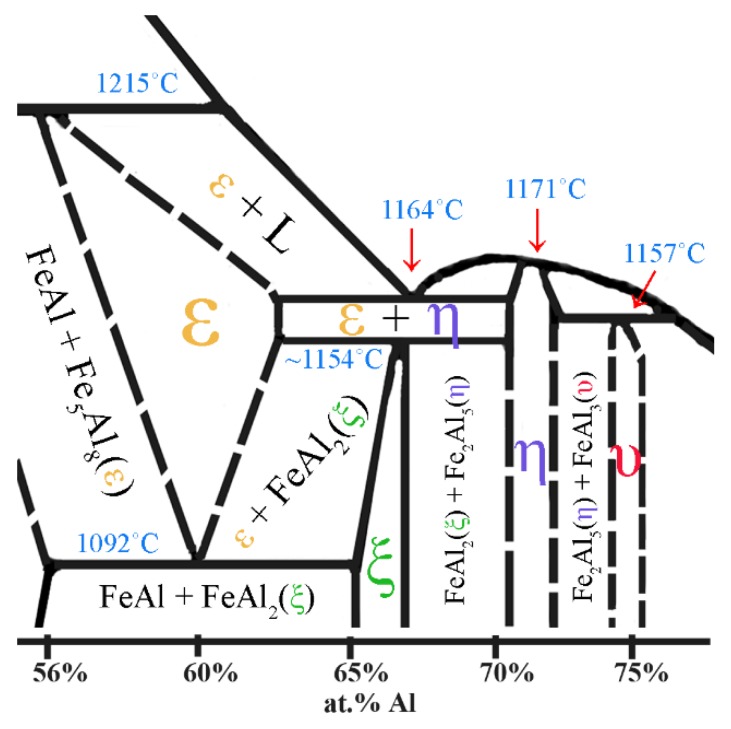
The part of Fe-Al phase binary diagram presenting the area of formation and transformations of Al-rich phases (made on the base of [15,16,17,18,19,20]).

**Figure 4:**
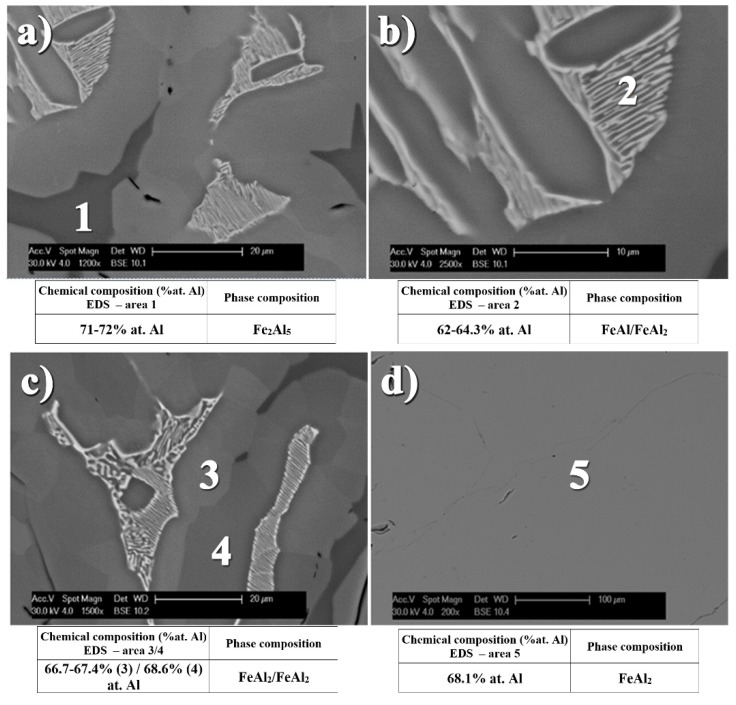
Inhomogeneous phase microstructure of material form Fe-Al system after crystallization (**a**–**c**) and homogenization to FeAl_2_ phase, as the result of annealing at temperature 1050 °C for 100 h (**d**).

Similar effects during melting and casting of Fe-Al alloys were also observed by Hirose *et al.* [39] and Gąsior *et al.* [40]. The low symmetry of the arrangement of the elementary cells of these phases, as reflected in the impeded phenomena of diffusion of the atoms of components, causes differences in the structure and chemical composition. This effect takes place during crystallization [39], which is substantially depended on the method and rate of cooling or subsequent thermal treatment [40]. In addition, possible fluctuations in the chemical composition of the metal liquid in conjunction with the temperature of the peritectic ε (Fe_5_Al_8_) transition (1215 °C) being higher than in the case of the eutectic ε + Fe_2_Al_5_ transformation at 1164 °C (Figure 3), lead to formation of the three-phase structure (Figure 4c). It is despite the fact that aluminum content provides the FeAl_2_ formation.

The performed microanalysis of the chemical composition in the areas of the various phase components formed in the investigated samples allows describing the sequence of phase transformation occurring in these alloys. During the cooling of the crystallized eutectic mixture (ε + Fe_2_Al_5_) with the peritectic ε, a successive phase transition takes place very quickly leading to the formation of the FeAl_2_ peritectoid at temperature about 1154 °C. Afterward at the temperature of 1092 °C the ε peritectic transforms into the eutectoid FeAl + FeAl_2_ mixture as a result of the eutectoid reaction. The difficult diffusion of atoms formed in the low-symmetry structures is also a likely cause of that differences in the content of aluminum in the locally formed FeAl_2_ phase which contains 66.7–68.1 at% of aluminum. Whenever the eutectoid FeAl + FeAl_2_ mixture resulting from the ε (Fe_5_Al_8_) phase is characterized by the aluminum content at the level of 62–64.3 at%. Whereas the congruent Fe_2_Al_5_ phase, crystallizing at the temperature of 1171 °C, comprises 71–72 at% of this element. This multi-phase structure of the assumed total aluminum content of 68 at% after 100 h of annealing at 1050 °C is remodeled by diffusion into a single-phase material corresponding with its chemical composition to the FeAl_2_ Al-rich phase (Figure 4d).

**Figure 5:**
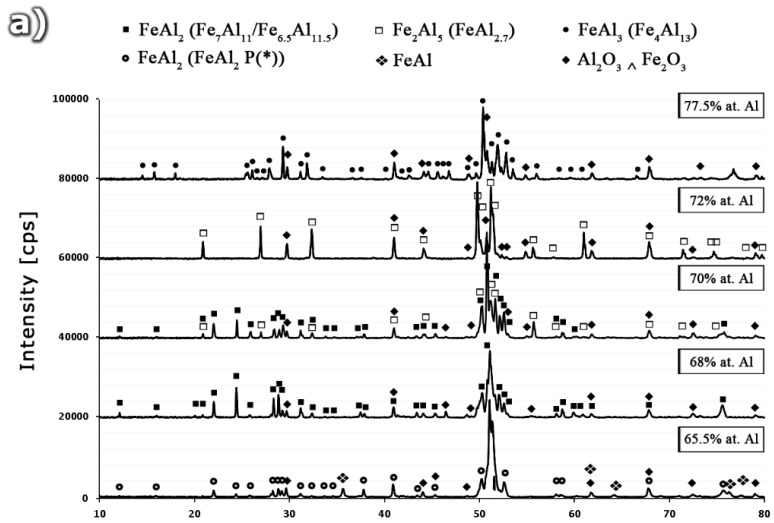

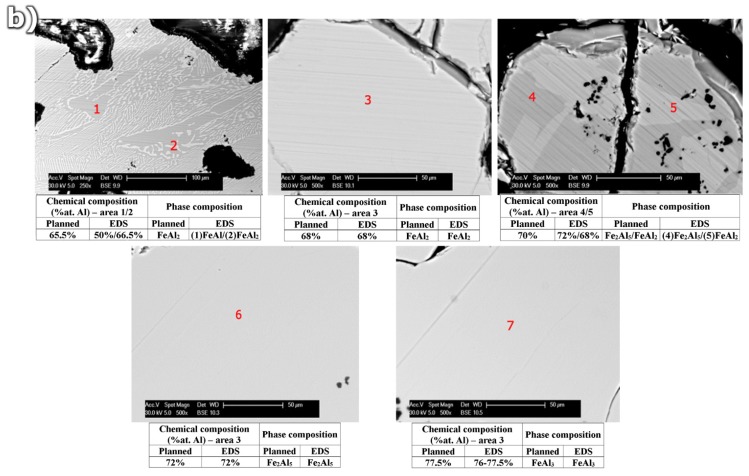
(**a**) The XRD patterns and (**b**) microstructure of alloys form Fe-Al system for various Al content 65.5 at%, 68 at%, 70 at%, 72 at% and 77.5 at%, as casted and annealed at temperature of 1050 °C for 100 h.

The performed X-ray phase analysis confirmed the results of EDS analysis, allowing for the identification of the single-phase structure obtained by homogenizing the crystallized ingots assuming the chemical composition of the Al-rich alloys, respectively, FeAl_2_—68 at% Al, Fe_2_Al_5_—72 at% Al, and FeAl_3_—77.5 at% Al (Figure 5).

**Figure 6:**
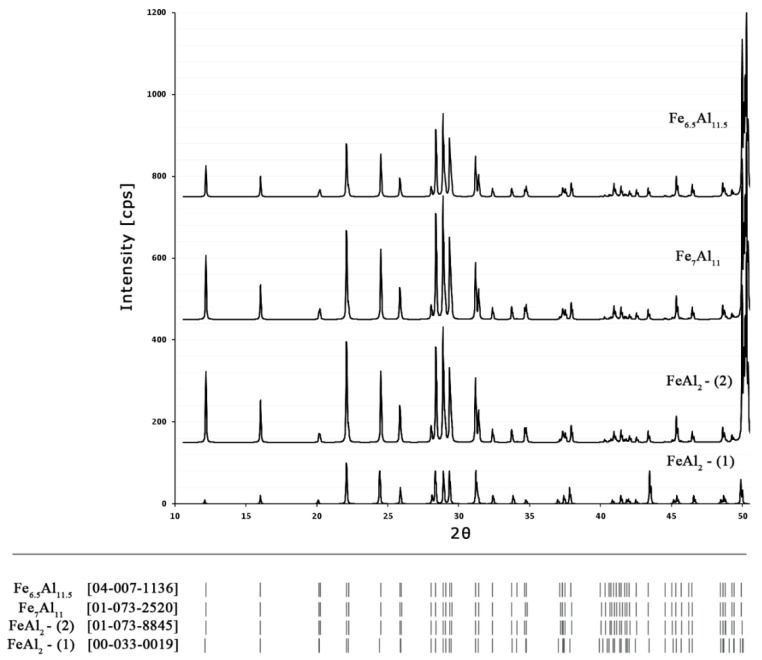
Different PDF files for FeAl_2_ phase with visible differences of diffraction peak positions and their intensity.

The XRD patterns for samples with 70 at% consist of peaks coming from a mixture of FeAl_2_ + Fe_2_Al_5_ phases, what is in agreement with the equilibrium system (Figure 2), as proposed by Kubaschewski [15] and others [17,18,19,20]. Whereas, the alloy containing 65.5 at% Al was identified as a mixture of FeAl + FeAl_2_. It should be noted that in the case of the sample containing 65.5 at% Al, diffraction peaks are most closely related to the data included in the PDF file (00-033-0019) [41] described as FeAl_2_. In the other samples (identified by EDS as FeAl_2_) with aluminum content increasing to 68 and 70 at%, the identification of peaks allows for their assignment to Fe_7_Al_11_ (01-073-2520) [42] and Fe_6.5_Al_11.5_ (04-007-1136), respectively [42]. The possibility of assigning the reflections originating from the same phase with slightly different aluminum content to different PDF files of different position, different intensity and different symmetry of reflections (Figure 6) is an indirect proof of the need to arrive at a clear crystallographic description of the phase, dependent on both the content of the constituent elements and the temperature [40,41,42,43,44,45,46]. Such work, involving the indexing of diffraction reflections from the obtained from Al-rich phases, including Fe_2_Al_5_ and FeAl_3_, using different methods (e.g., DICVOL66, ITO, N-TREOR), will be discussed in a separate publication.

### 2.2. Mechanical Properties Test

**Figure 7:**
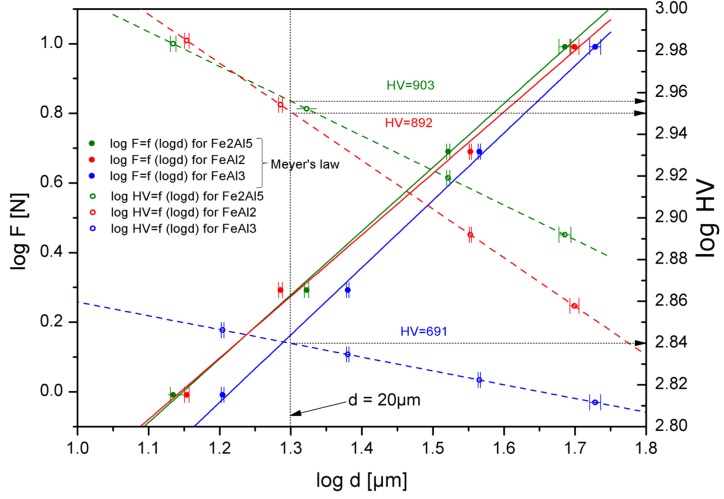
Hardness of Al-rich phases determined for hypothetical diagonal of indentation, which equals 20 μm.

Ambiguous, and above all, very few structural descriptions of the analyzed Al-rich FeAl_2_, Fe_2_Al_5_ and FeAl_3_ phases are also the reason for the small interest on the utility (structural or functional) of alloys containing these phases, and thus the lack of information about their properties, mostly mechanical. The literature only provides the hardness values of the Al-rich intermetallic phases, which are depended on the applied load, the method of preparation, the chemical composition and the macroscopic geometrical dimensions [24,25,26,27,28,29,39,40]. The intermetallic hardness value exists in the wide range of value, respectively, 900–1050 HV for FeAl_2_, 950–1100 HV for Fe_2_Al_5_ and 800–980 HV for FeAl_3_ [25,26,27,28]. Such wide ranges of hardness for structures with a narrow range of chemical composition, which are not subjected to allotrope changes, are highly ambiguous. Therefore, in order to mutually compare the hardness of the Al-rich phases produced by means of melting and casting, undergoing homogenization, their hardness was determined using the Mayer’s law and the law of variable hardness for the hypothetical diagonal print of 20 μm (Figure 7) [30].

The values obtained, measured on the phase-homogeneous ingots with a diameter of 30 mm and the height of 100 mm are noticeably lower, especially in the case of the FeAl_3_ phase than the literature data and are, respectively, 892 ± 6 HV for FeAl_2_, 903 ± 7 HV for Fe_2_Al_5_, and 691 ± 5 HV for FeAl_3_.

**Figure 8:**
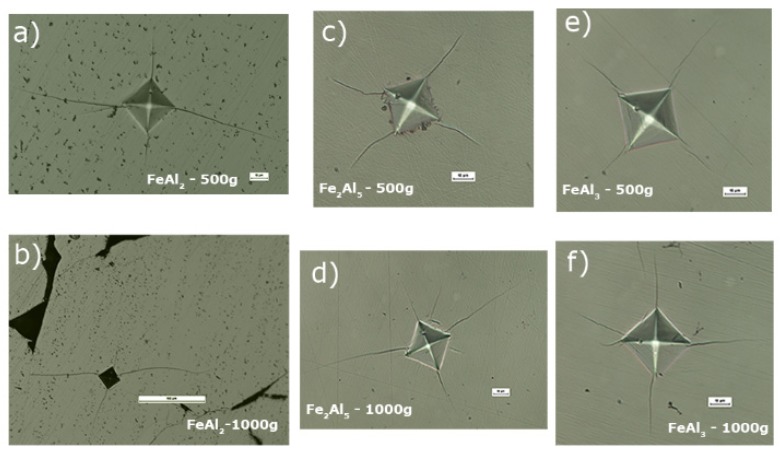
Indentations after Vickers hardness tests with propagating cracks observed for FeAl_2_ (**a**,**b**); Fe_2_Al_5_ (**c**,**d**); and FeAl_3_ (**e**,**f**) phases.

The microhardness measurements, in particular the clear and measurable cracks propagating from the corner prints (Figure 8) made it possible to assess the mechanical properties of the analyzed subsequent Al-rich phases form the Fe-Al system, in particular:

Stress intensity factor K_1C_ [47,48]:

K_1C_ = 0.0937 × [H_0_(P − P_0_)/4l]^1/2^ (1)

where H_0_—Vickers hardness (load-independent microhardness); P—Indentation load; P_0_—threshold indentation load for cracking; l—The length of the diagonal indentation.

Fragility factor I_b_ [49]:

I_b_ = HV/K_1C_ (2)

where HV—Vickers hardness; K_1C_—Stress intensity factor.

The threshold force of the indenter P* [49]:

P* = 1.6 × 10^4^ × K_1C_ (K_1C_/HV)^3^ (3)

where HV—Vickers hardness; K_1C_—Stress intensity factor.

Plastic deformation zone radius b [49]:

b = 0.69 × a × (E/HV)^0.5^ (4)

where a—The length of the crack; HV—Vickers hardness; E—Young’s modulus.

**Figure 9:**
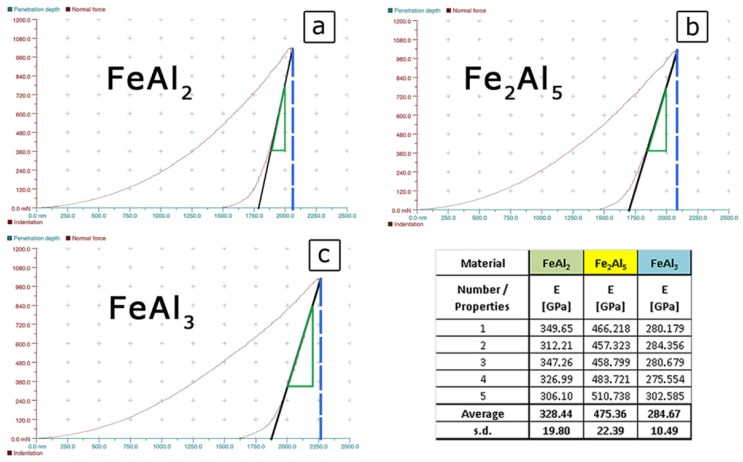
The results of Young’s modulus measurement for FeAl_2_ (**a**); Fe_2_Al_5_ (**b**); and FeAl_3_ (**c**) phases obtained by instrumented indentation.

However, for the quantitative assessment of these parameters, it is necessary to know the basic parameter characteristic for each type of material, namely, the Young’s modulus. The value of this parameter was determined by the nano-indentation Vickers indenter, assuming a constant Poisson’s ratio at the level of ν = 0.3 [29,50,51,52,53]. In this method, the value of the Young’s modulus is defined as the value of the slope of the tangent to the force recorded during the unloading the indenter (Figure 9) [30,48,49].

The obtained values are respectively: 328 ± 20 GPa for FeAl_2_, 475 ± 22 GPa for Fe_2_Al_5_ and 284 ± 10 GPa for FeAl_3_ which, at the spreading of the results at a level of 6%, indirectly proves high homogeneity, and thus a single-phase structure of the test samples.

The prints obtained when measuring hardness at the loads of 500 g and 1000 g, characterized by a grid of cracks occurring at the corners (Figure 9a–f), were used to determine the stress intensity factor which is a measure of the fracture toughness. The observation of the micro areas in the corners most likely indicates the Palmqvist cracks which are characteristic for ceramic materials or cermets *i.e.*, Si_3_N_4_, B_4_C, WC-Co, ZrO_2_ [53,54,55]. They are visible in the case of the FeAl_2_, FeAl_3_, and Fe_2_Al_5_ phases in the range of a load equal to 500 g (Figure 9a,c,f). Observed cracks take the form of short lines extending from the corners into the deformed zone. Moreover, in the case of 1000 g load (Figure 9b,d,e) well-visible additional lateral cracks of different lengths appear within the cracks’ area. There were, however, no branched cracks, typical for brittle ceramics, coming from the corners or sides of the recesses.

Indentation fracture toughness was calculated following reference [47] where the authors for the first time reported the existence of the compressive stresses core zone in the intermetallic compounds. Song and Varin [47] concluded that the most reasonable values of indentation fracture toughness for intermetallic compounds were obtained from the Palmqvist-type cracking using modified Shetty *et al.* model [48] modified by Song and Varin [47] for the indentation size effect (ISE).

The obtained values of this parameter for each of the studied iron aluminides amounted to: 0.592 ± 0.003 MPa/m^0.5^ for FeAl_2_, 0.817 ± 0.004 MPa/m^0.5^ for Fe_2_Al_5_ and 0.967 ± 0.005 MPa/m^0.5^ for FeAl_3_. The authors also drew attention to the dependence [49] determining the fragility “I_b_” factor (2), which is the ratio of hardness, on the fracture toughness, specifying the theoretical nature of the observed cracks. The relationship expressing the threshold force of the “P*” indenter (3) was also determined. This threshold force is the measure of cleavage allowing designing ceramic materials, *i.e.*, to control the level of strength of materials by controlling the hardness of the material or fracture toughness, for example, by introducing particulates impeding the development of cracks. The values for both of the factors are comparable to the values obtained for alundum ceramics (Al_2_O_3_) and are as follows: 1.40 ± 0.020 μm^−0.5^ for FeAl_2_, 0.92 ± 0.016 μm^0.5^ for Fe_2_Al_5_ and 0.70 ± 0.011 μm^−0.5^ for FeAl_3_ in the case of the fragility factor. In contrast, the threshold indentation force for FeAl_2_ is 3.4 ± 0.2 N, Fe_2_Al_5_—16.3 ± 0.9 N and for FeAl_3_—43.9 ± 2.2 N.

On the basis of the measurements and microscopic observations the “b” plastic deformation zone radius (4) was also evaluated. Usually, the main and side cracks run through it, and the zone is characterized by significant movements of the material and the large amount of short microcracks. Its size depends primarily on the brittleness of the material and is determined by the ratio of the hardness to the Young’s modulus or the ratio of the hardness to the values of the fracture toughness. Therefore it is associated with the development of cracks as a function of the size of the load, which determines the type of observed cracks. The highest value of this parameter was found for FeAl_2_—81.1 ± 0.9 μm, then for FeAl_3_—75.4 ± 0.7 μm and the radius of the plastic deformation zone for Fe_2_Al_5_ was estimated at 62.2 ± 0.9 microns.

### 2.3. Discussion

**Figure 10:**
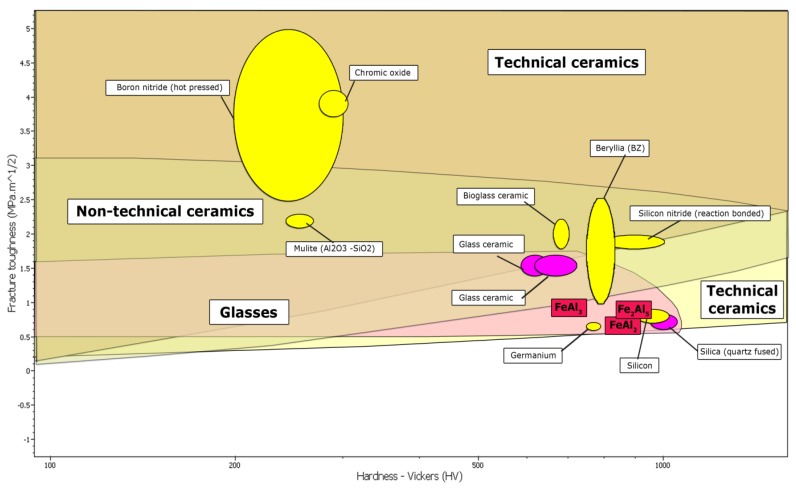
The comparison of mechanical properties for Al-rich FeAl_2_, Fe_2_Al_5_ and FeAl_3_ phases with the others materials.

Difficulties with the correct and unambiguous property determination of the FeAl_2_, Fe_2_Al_5_ and FeAl_3_ phases are mainly due to a slight difference in the aluminum concentration from FeAl_1.76_ to FeAl_3.25_. However, the difference in the aluminum concentration in conjunction with the low symmetry of the crystal arrangement of these phases and similar enthalpy of formation [40], determines substantially small, but measurable differences in the size of their strength parameters. Therefore, in order to obtain single-phase Al-rich structures, the alloys with specific and a very narrow range of chemical compositions, using the long-term homogenization process following the melting and casting, must be fabricated [29,52]. The lack of order and the semi-metallic [24] spin-glass [31] nature, also determined as quasi-amorphous [46], based on the nature of the crystal structure of these phases and the cardinality of atoms, put into question the applicability of the FeAl_2_, Fe_2_Al_5_ and FeAl_3_ phases despite their recently relatively well-recognized thermal and magnetic properties [31,44,46]. However, on the other hand, the determined mechanical properties of the Al-rich phases of the Fe-Al system, related to the lack of order of the crystal arrangement [29,52] locate them on the borderline of technical ceramics, glasses, and composite materials which can include, inter alia, nitrides and oxides of aluminum, or titanium oxides and molybdenum and boron carbide (Figure 10) (Table 1).

The analysis of the technological processes and the sought of the Al-rich phases applications provide for the greatest prospect of their use in the area of welding and the production of heat-resistant barrier coatings [56,57]. However, the potential area of their application may be changed at the time of acquiring comprehensive knowledge of the structural and strength properties determining their possible applications.

**Table 1:** The comparison of mechanical properties for Al-rich FeAl_2_, Fe_2_Al_5_ and FeAl_3_ phases with the others materials.

Material/Properties	HV [GPa]	E [GPa]	V [-]	K_1C_ [MPa/m^0.5^]	Ib [μm^−0.5^]	P* [N]	b [μm]	ref.
Diament	81	1000–1200	0.07–0.2	5.3	15.00	0.02	-	[49,54,55]
Al_2_O_3_	14–18	360–420	0.22–0.25	3–5	4.00	1.0	-	[49,54,55]
SiC	18–25	340–450	0.18–0.19	3	7.20	0.13	-	[49,54,55]
Steel	1.6–5.0	190–210	0.240–0.300	50–210	0.014	0.1 × 10^9^	-	[49,54,55]
Cu	0.1–0.8	100–124	0.310–0.340	100	0.008	3 × 10^9^	-	[49,54,55]
Al	0.1–0.4	69–71	0.260–0.360	350	0.001	4 × 10^12^	-	[49,54,55]
WC-Co	12–20	540–610	0.280–0.300	7–28	1.10	170	-	[54,58]
NiAl	2.7–5.3	100–310	0.23–0.45	4–6	0.50	110	-	[58,59,60]
Ni_3_Al	3.5–4.5	100–300	0.2–0.35	30–40	0.10	14 × 10^4^	-	[61,62]
Fe_3_Al	2.5–3.5	150	0.290–0.400	25–35	0.12	30 × 10^4^	-	[50,51,52,53]
FeAl	4–5.2	260	0.300–0.310	8–15	2.88	2875	-	[50,51,52,53,54,55,56,57]
FeAl_2_	9–10.5	475	0.300	3.88	2.71	4	81	[this study]
Fe_2_Al_5_	9.5–11	284	0.300	5.17	2.13	9	62	[this study]
FeAl_3_	8–9.8	328	0.300	4.92	1.99	13	75	[this study]

## 3. Experimental Section

In order to characterize the mechanical properties of the Al-rich phases, it was necessary to determine the content of aluminum allowing independent occurrence of these phases, without the possible (according to the equilibrium system (Figure 2)) presence of crystal mixtures. The problem has been solved by fabrication of the sample sets with various Al-content in the range from 56 at% (FeAl_3_ + FeAl_2_) to 80 at% (FeAl_3_ + Al(Fe)), using powder metallurgy of elementary components of iron and aluminum. Technically pure iron and aluminum powders were used for green body fabrication with a diameter of 20 mm and a height 10 mm, using the technique of uniaxial compression at ambient temperature under the pressure of 700 MPa. Then the samples were sintered at 1100 °C for approx. 2 h. The sintered material was homogenized at 1050 °C for 24 h in a vacuum after prior flushing the chamber with argon. The samples with single-phase structure were used to specify the aluminum content determining the presence of only one particular Al-rich phase. Further study of the mechanical properties of single-phase intermetallic alloys obtained by powder metallurgy was impossible due to the inherent presence of a structure made of sinters of elementary powders of oxide phases.

To investigate mechanical properties of single-phase alloys with chemical composition evaluated during sintering process, the other set of samples were made by melting and casting in a vacuum induction melting furnace Balzers VSG10. Then these samples were homogenized by annealing at 1200 °C for 100 h.

The samples obtained, both using powder metallurgy and melting and casting, were subjected to structural analysis (SEM, EDS) on the 3D Quanta FEG Dual Beam microscope. The phase identification was carried out using Rigaku Ultima IV diffractometer with cobalt target (*i.e.*, monochromatic radiation with a wavelength of 0.17889 nm was used). The analysis was conducted within the 2θ range of 20°–70°, at a scanning speed of 0.02°/min.

The final samples of single-phase structure obtained by melting and casting were used for the measurement of the microhardness carried out with Shimadzu type M Microhardness Tester at loads of 100 g, 500 g, 1000 g, in agreement with variable hardness law, for 10 s. Using the Nanoindentation Tester NHT Young’s modulus was determined for the Al-rich phases by analyzing changes in the load (500 g and 1000 g) with a change in the penetration depth of the indenter.

The micro-cracks observed in the corners of indentations were used to determine the critical stress intensity factor K_1C_, which is a measure of the fracture toughness [30,48,49,54,55,56,57,58,59,60,61,62]. The collected information allowed us to compare the properties of the obtained Al-rich single-phases intermetallic alloys with other structural intermetallics, brittle ceramic materials and the classic construction materials.

## 4. Conclusions

The results of microstructure investigation, in the area of Al-rich phases presence, showed the discrepancy of results in the phase identification already published. Moreover, due to realized structure analysis and observed phase transformation, the authors suggest different Al concentration for FeAl_2_, Fe_2_Al_5_ and FeAl_3_ phases.

The hardness, Young’s modulus and fracture toughness results of homogeneous intermetallic materials, such as FeAl_2_ (Fe_6.5_Al_11.5_), Fe_2_Al_5_ (FeAl_2.7_) and FeAl_3_ (Fe_4_Al_13_), are in good agreement with proposed phase transformation and phase identification.

On the base of obtained results, the authors suggest that the problem with Al-rich intermetallic phases implementation is not related to the luck of potential application. It is rather connected with rather poor knowledge about their structure and mechanical properties, which still require further investigation. 
